# Modeling Disease Progression: Angiotensin II Indirectly Inhibits Nitric Oxide Production via ADMA Accumulation in Spontaneously Hypertensive Rats

**DOI:** 10.3389/fphys.2016.00555

**Published:** 2016-11-17

**Authors:** Haidong Wang, Hao Jiang, Haochen Liu, Xue Zhang, Guimei Ran, Hua He, Xiaoquan Liu

**Affiliations:** Center of Drug Metabolism and Pharmacokinetics, China Pharmaceutical UniversityNanjing, China

**Keywords:** angiotensin II, asymmetric dimethylarginine, disease progression modeling, hypertension, intensive blood-pressure control, nitric oxide, spontaneously hypertensive rat

## Abstract

Nitric oxide (NO) production impairment is involved in the onset and development of hypertension. Although NO production impairment in spontaneously hypertensive rat (SHR) has been reported in a variety of researches, the time course of this progressive procedure, as well as its relationship with asymmetric dimethylarginine (ADMA) and angiotensin II (Ang II), has not been quantified. The aim of this research is to establish a mechanism-based disease progression model to assess Ang II and ADMA's inhibition of NO production in SHR's disease progression with/without ramipril's intervention. SHR were randomly divided into three groups: one disease group (*n* = 8) and two treatment groups (*n* = 8 for each group): standard treatment group (receiving ramipril 2 mg/kg^*^day) and intensive treatment group (receiving ramipril 10 mg/kg^*^day). ADMA, Ang II, NO, and SBP were determined weekly. Intensive treatment with ramipril was found to have no further attenuation of plasma NO and ADMA than standard treatment beyond its significantly stronger antihypertensive effects. Four linked turnover models were developed to characterize the profiles of ADMA, Ang II, NO, and SBP during hypertensive disease progression with/without ramipril intervention. Our model described Ang II and ADMA's contribution to NO production impairment and their responses to ramipril treatment throughout the disease progression in SHR. Model simulations suggested that Ang II affected NO production mainly through inhibiting ADMA elimination rather than affecting nitric oxide synthase (NOS) directly.

## Introduction

**Figure 1:**
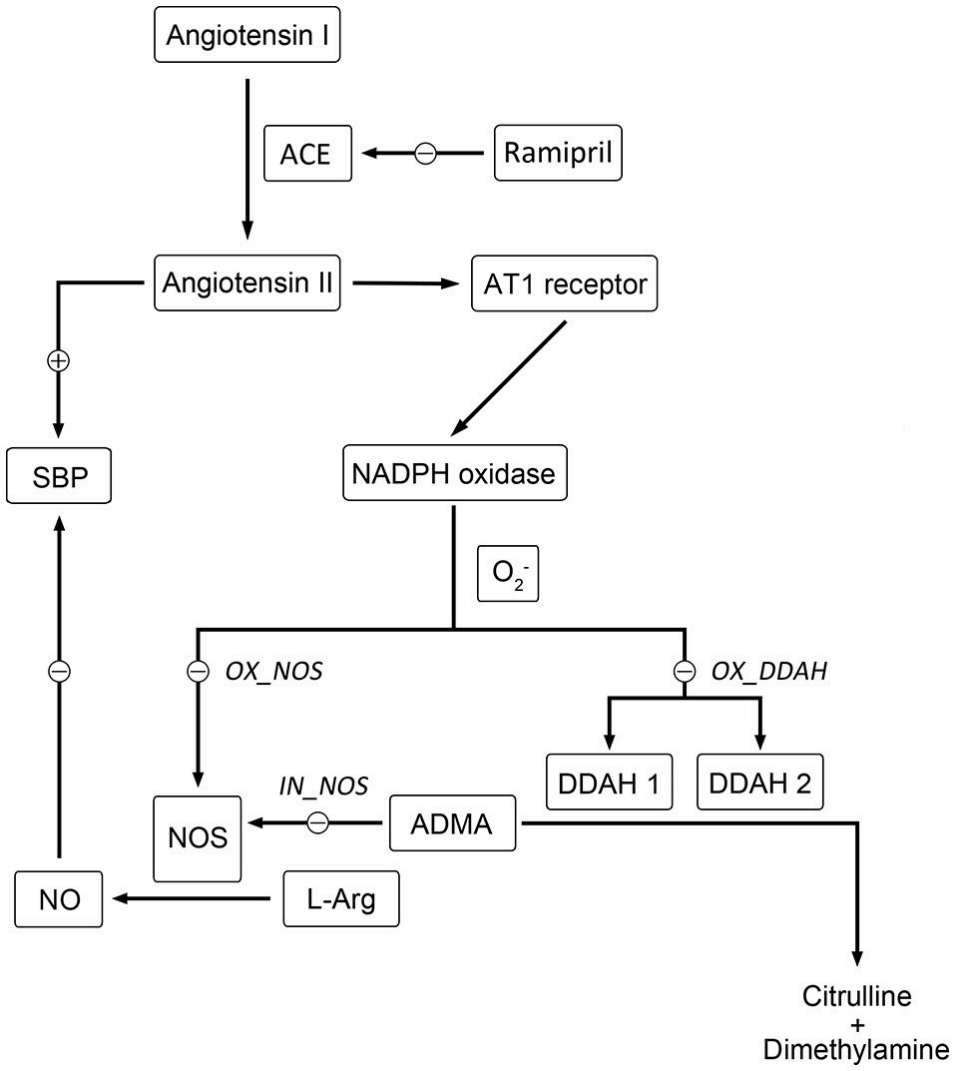
**Schematic diagram showing the mechanisms of Ang II affecting nitric oxide system**. Directly, Ang II-activated generation of free radicals inhibits the activity of NOS directly. Indirectly, the activity of DDAH is restricted by overproduced free radicals induced by Ang II, which leads to the accumulation of ADMA. Elevated level of ADMA inhibits the activity of NOS directly. The locations of simulated blockade are marked with corresponding disease factors (*IN_NOS, OX_DDAH*, and *OX_NOS*) beside.

Hypertension is a serious chronic disease that causes mortality and morbidity worldwide. A variety of pathophysiological mechanisms are involved in the genesis and development of hypertension (e.g., the activation of renin angiotensin system (RAS), impairment of nitric oxide synthase (NOS), oxidative stress, etc.; Hamza and Dyck, 2014). In hypertensive patients (Schulz et al., 2011) and rats (Landmesser et al., 2002; Mollnau et al., 2002), nitric oxide (NO) molecules are easily diminished by angiotensin II (Ang II) mediated over production of reactive oxygen species (ROS), leading to a reduction of NO bioavailability and endothelial dysfunction. Besides, enhanced ROS could also reduce the activity of NOS through oxidizing tetrahydrobiopterin (BH_4_), the cofactor of NOS, causing NOS impairment and convert NOS to superoxide generators, thus creating a vicious cycle (Baylis, 2012; Roe and Ren, 2012; Su, 2015). Although the end products of NO, nitrite and nitrate, could not reflect NO bioavailability, plasma nitrite, and nitrate together (NO_x_) has been widely used as an index of NO formation and break down, reflecting NOS activity indirectly (Zeballos et al., 1995; Jungersten et al., 1996). In addition, it is also suggested that urinary NO_x_ could not be used as a truly quantitative indicator of NO production (Baylis and Vallance, 1998), since NO might also be excreted through expired air or as other end products. Therefore, plasma NO_x_ was selected to be the indicator of NO production in this work. On the other hand, asymmetric dimethyl arginine (ADMA) plays an important role in bridging Ang II and NO. Elevated plasma ADMA level has been widely reported in hypertensive patients (Surdacki et al., 1999) and in SHR (Ghiadoni et al., 2007; Tain et al., 2011). According to previous studies, the major removal of ADMA is provided with dimethylarginine dimethylaminohydrolase (DDAH; Baylis, 2012). Hence, the activity of DDAH would affect the level of ADMA predominantly. As shown in Figure 1, the activity of both isoforms of DDAH suffers from an intensive inhibition by Ang II-mediated ROS generation (Palm et al., 2007; Baylis, 2012). Since DDAH provides the majority of ADMA removal (Baylis, 2012), inhibition of DDAH activity would lead to accumulation of ADMA *in vivo*, which subsequently affects the activity of NOS. Since these markers are closely related to each other and contribute greatly to hypertension, assessing the longitudinal time course of these markers might provide a better understanding of NO production impairment in hypertension disease progression of SHR.

The idea of modeling disease progression has been widely used in chronic diseases, for example diabetes (Cao et al., 2011; Gao et al., 2011), Parkinson's disease (Vu et al., 2012), Alzheimer's disease (Zhou et al., 2013), and hypertension (Zhou et al., 2012). Describing disease progression with responses to treatment in a quantitative way makes predicting clinical outcome events possible, which is especially essential in long-term progressive diseases with poor prognosis (Holford, 2015). On the other hand, model-based evaluation of disease progression provides insight into the mechanism as well as evaluation of drug effect on disease progression (Mould et al., 2007). In a previous study carried out with Zhou et al., a disease progression model was established for capturing the counter-balance relationship between Ang II and Ang-(1-7) in SHR (Zhou et al., 2012). The model satisfyingly described the two peptides' counter regulatory effects on blood pressure. In this paper, we aimed to offer a better understanding of Ang II and ADMA's contributions to NO production impairment in disease progression of SHR, with our proposed model.

Ramipril, an ACE (angiotensin converting enzyme) inhibitor, has been demonstrated to show cardiovascular protection in SHR beyond antihypertensive action (Linz et al., 1995; Gohlke et al., 1996). In addition, ramipril was also reported to ameliorate endothelial dysfunction, restore NOS impairment, and improve oxidative stress (Linz et al., 2003; Yilmaz et al., 2007). Therefore, ramipril was selected as a tool for modeling and validation, in order to gain more information about the relationship between elevated plasma Ang II level and NO production impairment. Two different doses were set up to investigate whether intensive treatment with ramipril could have more attenuation of NO production impairment dependently of intensified blood pressure control.

## Materials and methods

### Animals

Twenty-four 4-week-old male spontaneously hypertensive rats were purchased from Vital River Laboratory Animal Technology Co., Ltd. (Beijing, China). All rats were raised in 12-h light/12-h dark cycle environment and had free access to water and food. This study was approved by Ethics Committee for Animal Experimentation of China Pharmaceutical University. All efforts were made to minimize animal suffering.

### Materials

Ramipril was supplied by Kunshan Rotam Reddy Pharmaceutical Co., Ltd. (Kunshan, China). Systolic blood pressure was measured with ALC-NIBP (tail-cuff method) from ALCBIO (Shanghai, China). Iodine [^125^I] Angiotensin II Radioimmunoassay Kit was obtained from Beijing North Institute of Biological Technology (Beijing, China).

### Experimental design

All rats were acclimatized for 1 week. From the age of 5 week, 24 SHR were randomly assigned to three groups: one disease group and two ramipril treatment groups: standard treatment group (receiving ramipril 2 mg/kg/day) and intensive treatment group (receiving ramipril 10 mg/kg/day). Rats in two treatment groups were given ramipril by gavage at 9:00 AM every day from 18 to 21-week-age. SBP were measured weekly. Six hundred microliter of blood sample was collected via tail vein once a week with collection time fixed at 2:00 PM, blood samples were anticoagulated with EDTA and centrifuged at 4000 g for 15 min immediately. Plasma samples were aliquoted and stored at −80°C until analysis. At the age of 21 week, all rats were sacrificed by cervical dislocation.

### Blood pressure measurement

The protocol for blood pressure measurement was designed based on the method introduced in the works of Whitesall et al. (2004) and Kubota et al. (2006). During the first week, all rats were acclimated to restraint, tail-cuff inflation, and heating. Rats were placed in plastic restrainers with heating pad remaining at 33~34°C. The instrument (ALC-NIBP, ALCBIO; Shanghai, China) automatically takes ten 30-s measurements. The values of systolic blood pressure were recorded when more than five consecutive stable readings were available. The highest and lowest readings were discarded, and the remaining readings were averaged for one data point.

### ADMA, NO, and Ang II assays

Plasma asymmetric dimethylarginine (ADMA) was measured using an HPLC-MS/MS method introduced by He (He et al., 2013). Plasma nitric oxide (NO) is determined by measuring the stable end products, nitrite and nitrate, which is described in the work of Moshage (Moshage et al., 1995). Plasma angiotensin II (Ang II) was measured using radioimmunoassay with commercial kit obtained from Beijing North Institute of Biological Technology (Beijing, China).

### Disease progression model

**Figure 2:**
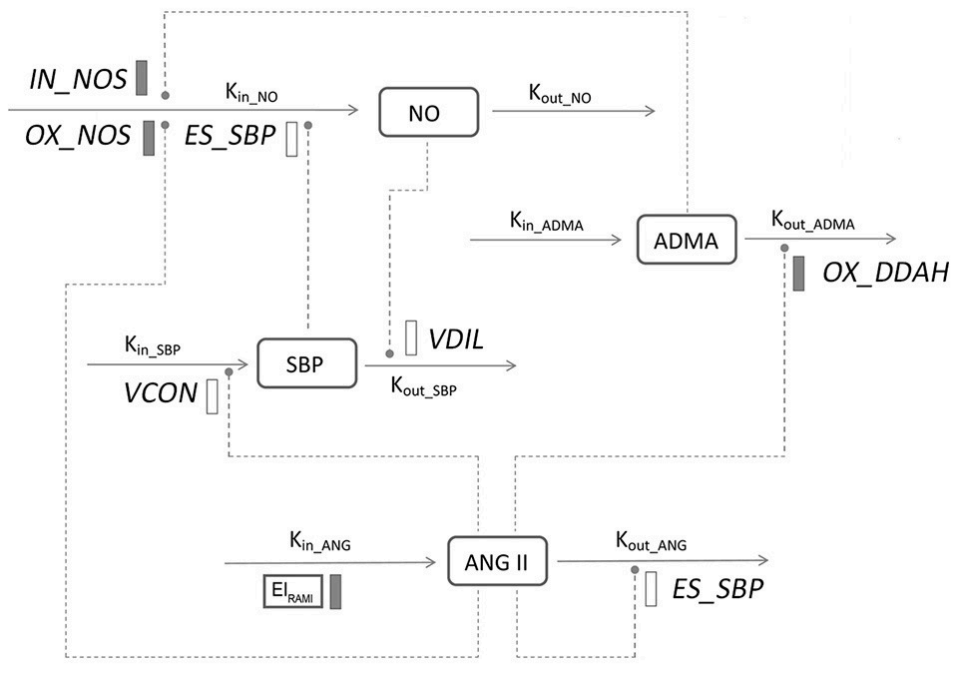
**Model structure defining the interactions between ADMA, Ang II, NO, and SBP during hypertension progression**. Symbols and parameters are defined under Materials and Methods and in Table 1. Lines with arrows indicate conversion to or turnover of the indicated factors. Dashed lines ending in closed circles indicate an action is exerted by the connected factors. Solid bars indicate inhibiting effects, open bars indicate stimulating effects.

The general structure of the disease progression model is shown in Figure 2. Basically, the model was composed with three components: one defined the natural disease progression in SHR without treatment as disease model; two of which described ameliorated hypertensive disease progression with two different doses of ramipril intervention as treatment model. Four turnovers were applied for describing the dynamics of plasma Ang II, plasma ADMA, plasma NO, and SBP, which are represented by the following four equations:

(1) $$\begin{array}{l} \frac{dC_{ANG}}{dt}=k_{in\_ANG}\left(1/\left(EI_{RAMI}\cdot \text{exp}\left(DOSE\right)\right)\right)\\ -\;k_{out\_ANG}\cdot C_{ANG}\cdot \left(1+ES_{ANG}\cdot C_{ANG}\right) \end{array}$$

(2) $$\begin{array}{l} \frac{dC_{ADMA}}{dt}=k_{in\_ADMA}-k_{out\_ADMA}\\ \cdot C_{ADMA}\left(1-IA_{ANG}\left[m\right]\cdot C_{ANG}\right) \end{array}$$

(3) $$\begin{array}{l} \frac{dC_{NO}}{dt}=k_{in\_NO}(1-IN_{ADMA}\left[n\right]\cdot C_{ADMA}-IN_{ANG}\left[o\right]\\ \;\cdot C_{ANG}+ES_{SBP}\cdot C_{SBP})-k_{out\_NO}\cdot C_{NO} \end{array}$$

(4) $$\begin{array}{l} \frac{dSBP}{dt}=k_{in\_SBP}\left(1+ES_{ANG}\left[p\right]\cdot C_{ANG}\right)-k_{out\_SBP}\\ \cdot \left(1+ES_{NO}\left[q\right]\right)\cdot SBP \end{array}$$

In which, *C*_*ANG*_, *C*_*ADMA*_, *C*_*NO*_, and *SBP* indicate plasma Ang II, ADMA, NO concentration, and SBP level, respectively. In this model, plasma Ang II was the marker that triggers the cascade of downstream reactions. Plasma Ang II is assumed to be formed at a zero-order constant rate (*k*_*in*_*ANG*_) and degraded by the first-order constant rate (*k*_*out*_*ANG*_). The degradation of Ang II is stimulated through a negative regulatory feedback loop (*ES*_*ANG*_; Zhou et al., 2012), which is characterized with a linear model represented with *ES*_*ANG*_. In two treatment groups, plasma Ang II level is subjected to an inhibitory effect from ramipril (*EI*_*RAMI*_), *EI*_*RAMI*_ is a drug-specific parameter. The dose of ramipril was indicated by parameter *DOSE*. *DOSE* was fixed at 0 when model was fitted in disease group, while at 2 or 10 in two treatment groups, respectively. ADMA is synthesized by Protein Arginine Methyltransferase (PRMT) and eliminated mainly through dimethylarginine dimethylaminohydrolase (DDAH) hydrolysis. Prior to occurrence of disease, plasma ADMA level remains at a relatively stable situation, which is represented by a zero-order constant rate (*k*_*in*_*ADMA*_) for production and a first-order constant rate (*k*_*out*_*ADMA*_) for elimination. According to the previously mentioned mechanism, elimination of ADMA is restrained with Ang II (*IA*_*ANG*_[*m*]).*IA*_*ANG*_[*m*] represents the inhibitory effect that Ang II exerts on DDAH, where *m* indicates the number of transit compartments that is applied to simulate the inhibition of ADMA elimination by Ang II. A zero-order constant rate *k*_*in*_*NO*_ and a first-order constant rate *k*_*out*_*NO*_ are used to describe the generation and degradation of plasma NO, respectively. In this system, activity of NOS is stimulated by elevated blood pressure according to previous research (Vaziri et al., 1998), which is simulated with a linear effect represented by parameter *ES*_*SBP*_. The suppression of NOS activity by plasma ADMA and plasma Ang II are described with two transduction procedures *IN*_*ADMA*_[*n*] and *IN*_*ANG*_[*o*], respectively. *n* and *o* indicate the number of the transit compartments that is required for describing the effects. In this model, systolic blood pressure is assumed to be input and output in zero-order rate (*k*_*in*_*SBP*_) and first-order rate (*k*_*out*_*SBP*_), respectively, where Ang II contributes to the climb of systolic blood pressure by causing vasoconstriction, while NO eases systolic blood pressure through vasodilation effect, which are represented with two separate series of transit compartments *ES*_*ANG*_[*p*] and *ES*_*NO*_[*q*].

### Ang II dynamics

At the beginning of the disease progression, plasma Ang II level is described with the equation below:

(5) $$\begin{array}{l} C_{ANG}\left(0\right)=\frac{k_{in\_ANG}}{k_{out\_ANG}} \end{array}$$

In this model, Ang II accumulation is assumed to be spontaneous and no other markers in this system would affect the procedure of accumulation.

### ADMA dynamics

Plasma ADMA was assumed to be maintained at a steady level before the initiation of the disease development, which is reflected with the equation below:

(6) $$\begin{array}{l} C_{ADMA}\left(0\right)=\frac{k_{in\_ADMA}}{k_{out\_ADMA}} \end{array}$$

The turnover of plasma ADMA was mainly affected by the activity of DDAH, which was inhibited by Ang II-induced ROS due to its high sensitivity to oxidative environment. This inhibitory effect was simulated by a series of transit compartments, which are represented by the following equations:

(7) $$\begin{array}{l} \frac{dIA_{ANG}\left(0\right)}{dt}=OX\_DDAH-kt_{1}\cdot IA_{ANG}\left(0\right)\\ \frac{dIA_{ANG}\left(1\right)}{dt}=kt_{1}\cdot IA_{ANG}\left(0\right)-kt_{1}\cdot IA_{ANG}\left(1\right)\\ \;\ldots \\ \frac{dIA_{ANG}\left(m\right)}{dt}=kt_{1}\cdot IA_{ANG}\left(m-1\right)-kt_{1}\cdot IA_{ANG}\left(m\right) \end{array}$$

This transduction effect was assumed to be initiated by a disease factor *OX*_*DDAH*, where *m* indicates the number of transit compartments that were applied to describe the inhibition of Ang II-mediated ROS on the elimination of ADMA; each transit compartment was connected by a turnover rate constant *kt*_1_. Different transit compartment numbers were evaluated to find a number that sufficiently captured the stimulation.

### NO dynamics

The initial plasma NO level is represented with the following equation:

(8) $$\begin{array}{l} C_{NO}\left(0\right)=\frac{k_{in\_NO}}{k_{out\_NO}} \end{array}$$

During disease progression, the turnover of NO was mediated by other three markers in this system. ADMA inhibits the synthesis of NO directly by competitive binding to NOS. A series of transit compartments was applied to describe this effect:

(9) $$\begin{array}{l} \frac{dIN_{ADMA}\left(0\right)}{dt}=IN\_NOS-kt_{2}\cdot IN_{ADMA}\left(0\right)\\ \frac{dIN_{ADMA}\left(1\right)}{dt}=kt_{2}\cdot IN_{ADMA}\left(0\right)-kt_{2}\cdot IN_{ADMA}\left(1\right)\\ \;\ldots \\ \frac{dIN_{ADMA}\left(n\right)}{dt}=kt_{2}\cdot IN_{ADMA}\left(n-1\right)-kt_{2}\cdot IN_{ADMA}\left(n\right) \end{array}$$

This transduction effect was assumed to be initiated by a disease factor *IN_NOS*, where *n* indicates the number of transit compartments that were applied to simulate the inhibitory effect of ADMA on the generation of NO; each transit compartment was connected by a turnover rate constant *kt*_2_. Different transit compartment numbers were evaluated to find a number that sufficiently captured the inhibitory effect from ADMA on NOS.

Ang II-induced generation of ROS would sharply cut down the activity of NOS. To mimic this effect, a series of transit compartments were utilized:

(10) $$\begin{array}{l} \frac{dIN_{ANG}\left(0\right)}{dt}=OX\_NOS-kt_{3}\cdot IN_{ANG}\left(0\right)\\ \frac{dIN_{ANG}\left(1\right)}{dt}=kt_{3}\cdot IN_{ANG}\left(0\right)-kt_{3}\cdot IN_{ANG}\left(1\right)\\ \;\ldots \\ \frac{dIN_{ANG}\left(o\right)}{dt}=kt_{3}\cdot IN_{ANG}\left(o-1\right)-kt_{3}\cdot IN_{ANG}\left(o\right) \end{array}$$

This transduction effect was assumed to be initiated by a disease factor *OX*_*NOS*, where *IN*_*ANG*_ (*o*) indicates the number of transit compartments that were applied to describe the inhibition from Ang II-induced ROS on the generation of NO; each transit compartment was connected by a turnover rate constant *kt*_3_. Different transit compartment numbers were evaluated to find a number that sufficiently captured the stimulation.

### SBP dynamics

SBP continues to climb during the growth of SHR till the age of 16–17 weeks. At the age of 5 weeks, the SBP of SHR is described by the equation below:

(11) $$\begin{array}{l} C_{SBP}\left(0\right)=\frac{k_{in\_SBP}}{k_{out\_SBP}} \end{array}$$

In the system we investigated, SBP was adjusted by Ang II and NO in a manner of counterbalance. Ang II exerted its pressor effect by causing vasoconstriction through binding to AT1 receptors. On the contrary, the pressor effect was being counterbalanced by vasodilator NO. This effect could be described through two series of transit compartments, which are represented by the following equations, respectively.

The first group of equations is proposed to represent vasopressor effect:

(12) $$\begin{array}{l} \frac{dES_{ANG}\left(0\right)}{dt}=VCON-kt_{4}\cdot ES_{ANG}\left(0\right)\\ \frac{dES_{ANG}\left(1\right)}{dt}=kt_{4}\cdot ES_{ANG}\left(0\right)-kt_{4}\cdot ES_{ANG}\left(1\right)\\ \;\ldots \\ \frac{dES_{ANG}\left(p\right)}{dt}=kt_{4}\cdot ES_{ANG}\left(p-1\right)-kt_{4}\cdot ES_{ANG}\left(p\right) \end{array}$$

*VCON* was assumed to be the initiative factor of vasoconstriction, *p* indicates the number of transit compartments that were applied to describe the vasoconstriction effect from Ang II; each transit compartment was connected by a turnover rate constant *kt*_4_. Different transit compartment numbers were evaluated to find a number that sufficiently captured the stimulation.

The vasodilation effect of NO was represented with the equations below:

(13) $$\begin{array}{l} \frac{dES_{NO}\left(0\right)}{dt}=VDIL-kt_{5}\cdot ES_{NO}\left(0\right)\\ \frac{dES_{NO}\left(1\right)}{dt}=kt_{5}\cdot ES_{NO}\left(0\right)-kt_{5}\cdot ES_{NO}\left(1\right)\\ \;\ldots \\ \frac{dES_{NO}\left(q\right)}{dt}=kt_{5}\cdot ES_{NO}\left(q-1\right)-kt_{5}\cdot ES_{NO}\left(q\right) \end{array}$$

The initiation of the vasodilation from NO was assumed to be initiated by *VDIL*, *q* indicates the number of transit compartments that were applied to describe the vasodilation effect of NO; each transit compartment was connected by a turnover rate constant *kt*_5_. Different transit compartment numbers were evaluated to find a number that sufficiently captured the stimulation.

### Modeling and simulation

The hypertensive disease progression combined with ramipril's effect was modeled using Phoenix 6.4 (CERTARA). The data from total 24 rats in the disease group and treatment groups were pooled together for baseline analysis in the initial 13 weeks. In the following 4 weeks, 8 rats in disease group were contributed continually for baseline modeling, and 8 rats in standard treatment group were used to estimate the drug effect parameter *EI*_*RAMI*_. Eight rats in intensive treatment group were used to validate the drug effect modeling. The validation was performed by visual predictive check (VPC). Estimates of parameters from the standard treatment group were used for the performance of VPC. The baseline parameters were accordingly fixed in drug effect estimation, assuming the baseline in treatment groups did not change evidently from the disease group. The dose of ramipril was represented with the parameter *DOSE. DOSE* was fixed at 0 in the disease group, while 2 and 10 in two treatment groups, respectively. Model evaluation was performed using non-parametric bootstrap analysis, introduced in previous research (Chen et al., 2009). Random draws of individual data from the original dataset was repeated 100 times. The stability of the final model was evaluated by comparing the model parameter estimates from the average values of new datasets with that obtained from the fit of the average values of original dataset.

Model simulations were conducted using mean estimates obtained from the model to observe the effects on plasma NO turnover throughout the disease progression from the three disease factors: *OX_DDAH, IN_NOS*, and *OX_NOS*. *OX_DDAH, IN_NOS*, or *OX_NOS* was fixed at zero in each simulation respectively, which simulates the blockade of such disease procedure.

## Results

### Dynamics of SBP, plasma Ang II, ADMA, and NO

**Figure 3:**
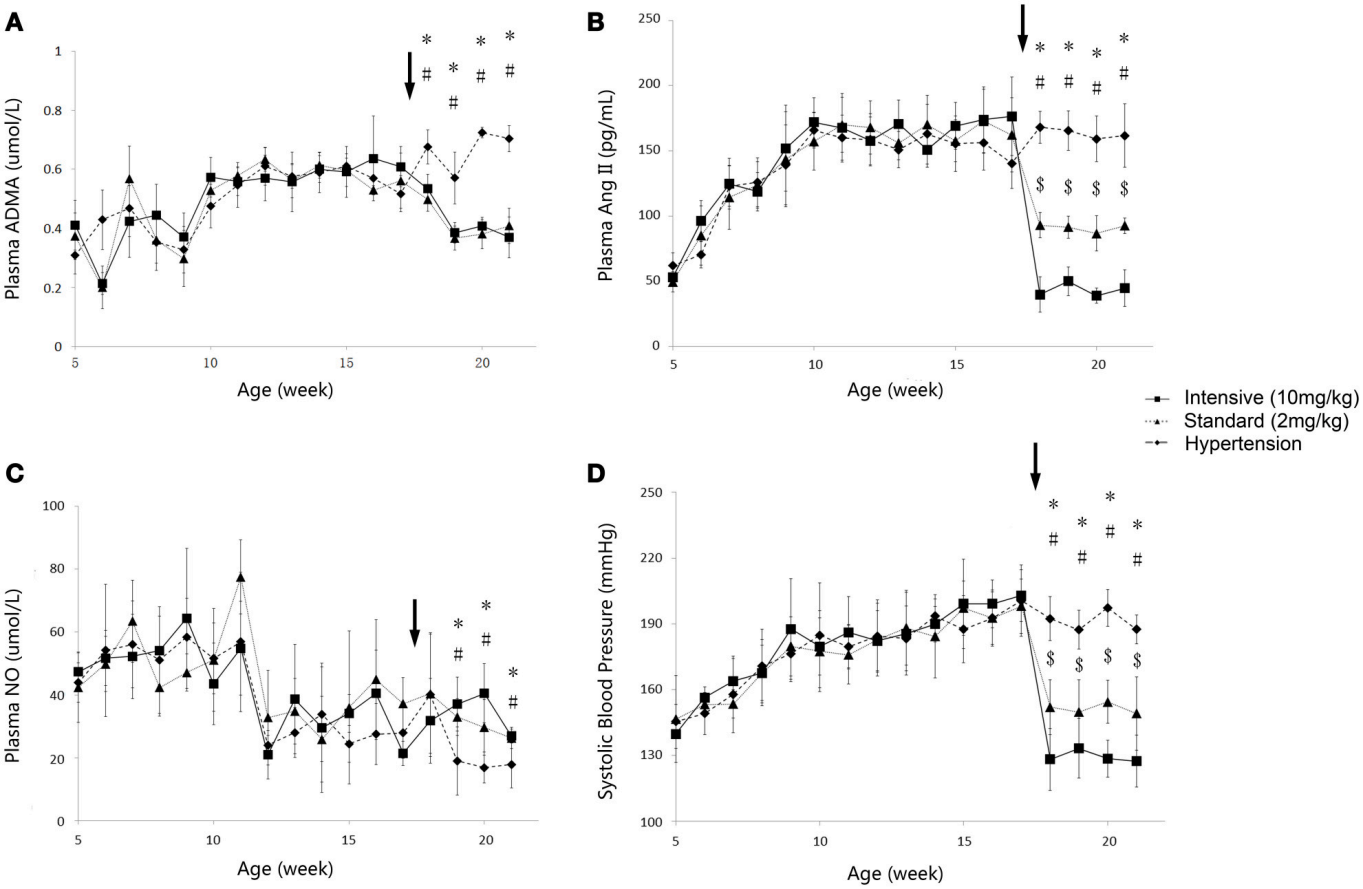
**Time course of plasma ADMA (A)**, Ang II **(B)**, NO**(C)** and SBP **(D)** progression in the disease group (solid diamonds), standard treatment group (2 mg/kg) (solid triangles), and intensive treatment group (10 mg/kg) (solid squares). Data are presented as mean ± *SD*. ^*^p < 0.05 (one way ANOVA): Disease group compares to standard treatment group (2 mg/kg). #*p* < 0.05 (one way ANOVA): Disease group compares to intensive treatment group (10 mg/kg). ^$^*p* < 0.05 (one way ANOVA): Standard treatment group (2 mg/kg) compares to intensive treatment group (10 mg/kg). The arrows point to the onset time of treatment.

The time course of plasma angiotensin II (Ang II), asymmetric dimethylarginine (ADMA), nitric oxide (NO) and systolic blood pressure (SBP) variations during 5 to 21-week-age are shown in Figure 3. In disease group, the change of SBP could be divided into three stages. At the first stage, SBP climbed quickly from under 140–180 mmHg during the first 4–5 weeks. SBP then grew to around 200 mmHg at the age of 17 weeks in a more slowly pace and remained at this level afterwards. Ramipril showed significant antihypertensive effect from the data of two treatment groups with a good dose-effect relationship. After 4 weeks' therapy, SBP reached 146.8 ± 13.44 mmHg in standard treatment group and 127.4 ± 11.9 mmHg in intensive treatment group, respectively. Time course of plasma Ang II level in all three groups of SHR went accordingly with the variation of SBP. Higher dose of ramipril also showed a stronger effect on plasma Ang II. Plasma ADMA, and NO did not vary sharply compared to SBP and plasma Ang II. Nevertheless, the accumulation of ADMA was observed in disease group. Plasma NO was maintained at a relatively higher level before a drop at the age of 12 weeks in all groups. Ramipril increased plasma NO during the 4-week therapy. It is worth mentioning that administration of 2 mg/kg ramipril successfully dragged SBP to 146.8 ± 13.44 mmHg, reversed the accumulation of plasma ADMA and increased plasma NO level, compared to disease group. However, a higher dose (10 mg/kg) of rampril with better antihypertensive effect (dragging SBP to 127.4 ± 11.9 mmHg) failed to exert further attenuation of plasma ADMA and NO.

### Disease progression model analysis

**Figure 4:**
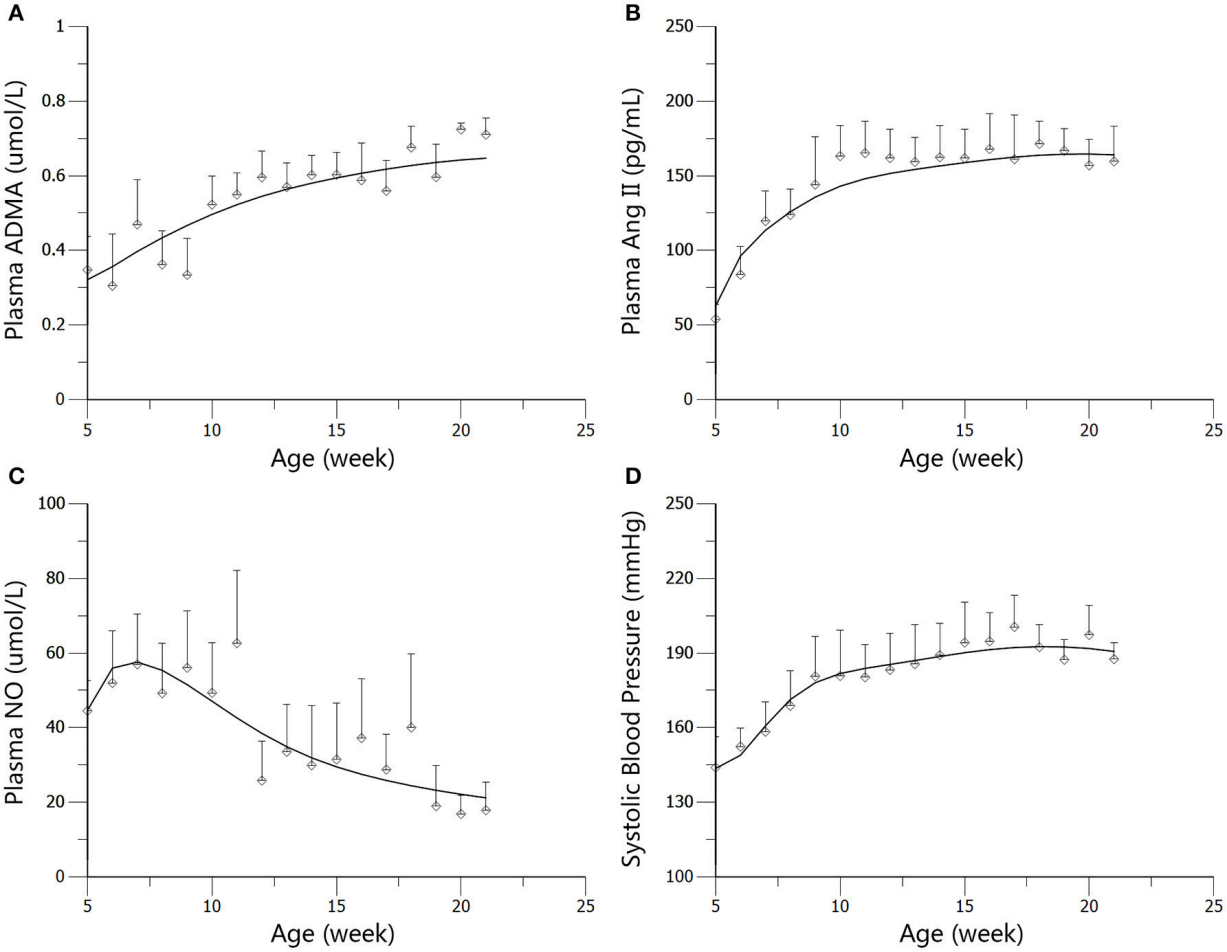
**Predicted and observed values for ADMA (A)**, Ang II **(B)**, NO **(C)**, and SBP **(D)** in the disease group. All observations are reported as Mean ± *SD* (open circles). The solid lines are the predicted values generated based on the original dataset.

**Figure 5:**
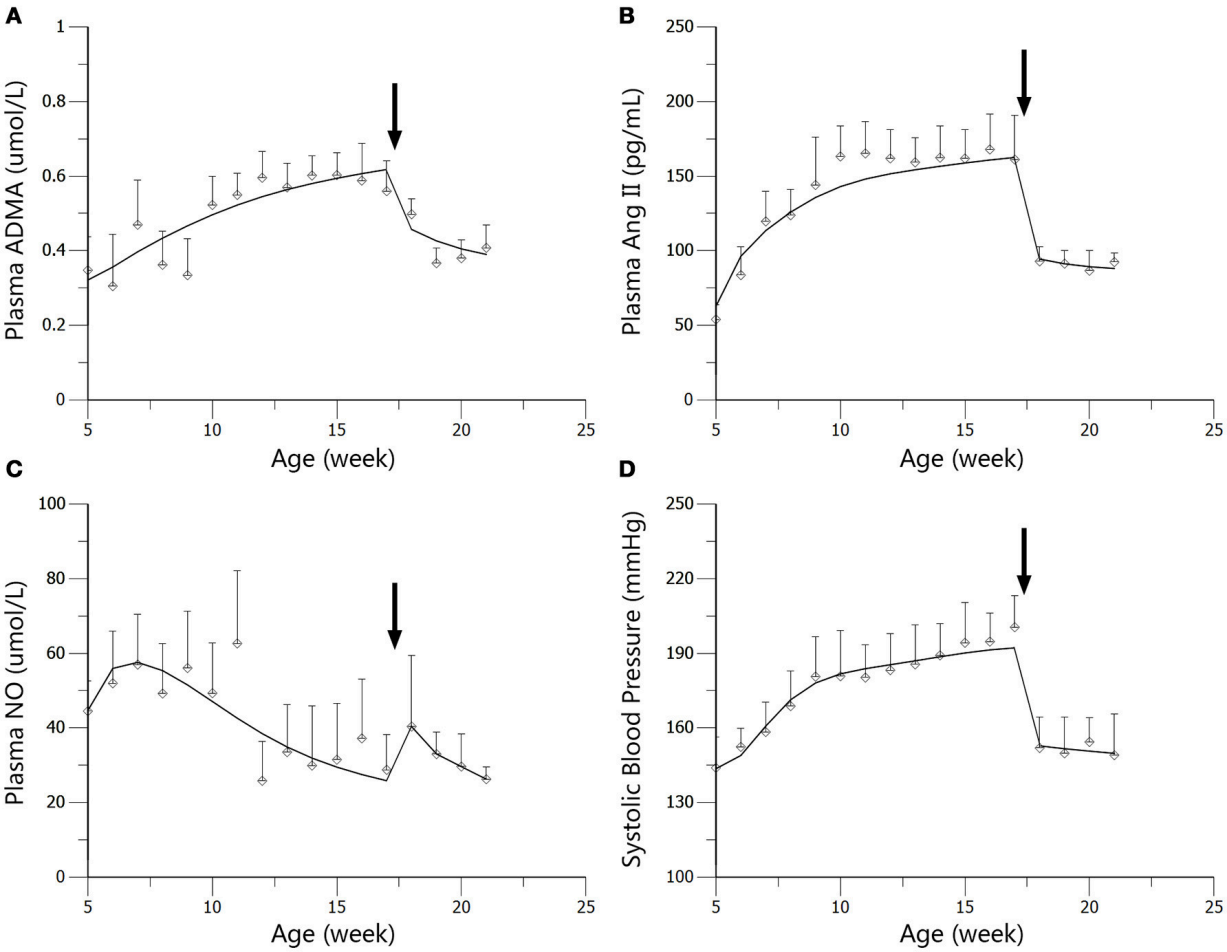
**Predicted and observed values for ADMA (A)**, Ang II **(B)**, NO **(C)**, and SBP **(D)** in the standard treatment group. All observations are reported as Mean ± *SD* (open circles). The solid lines are the predicted values generated based on the original dataset. The arrows point to the onset time of treatment.

**Figure 6:**
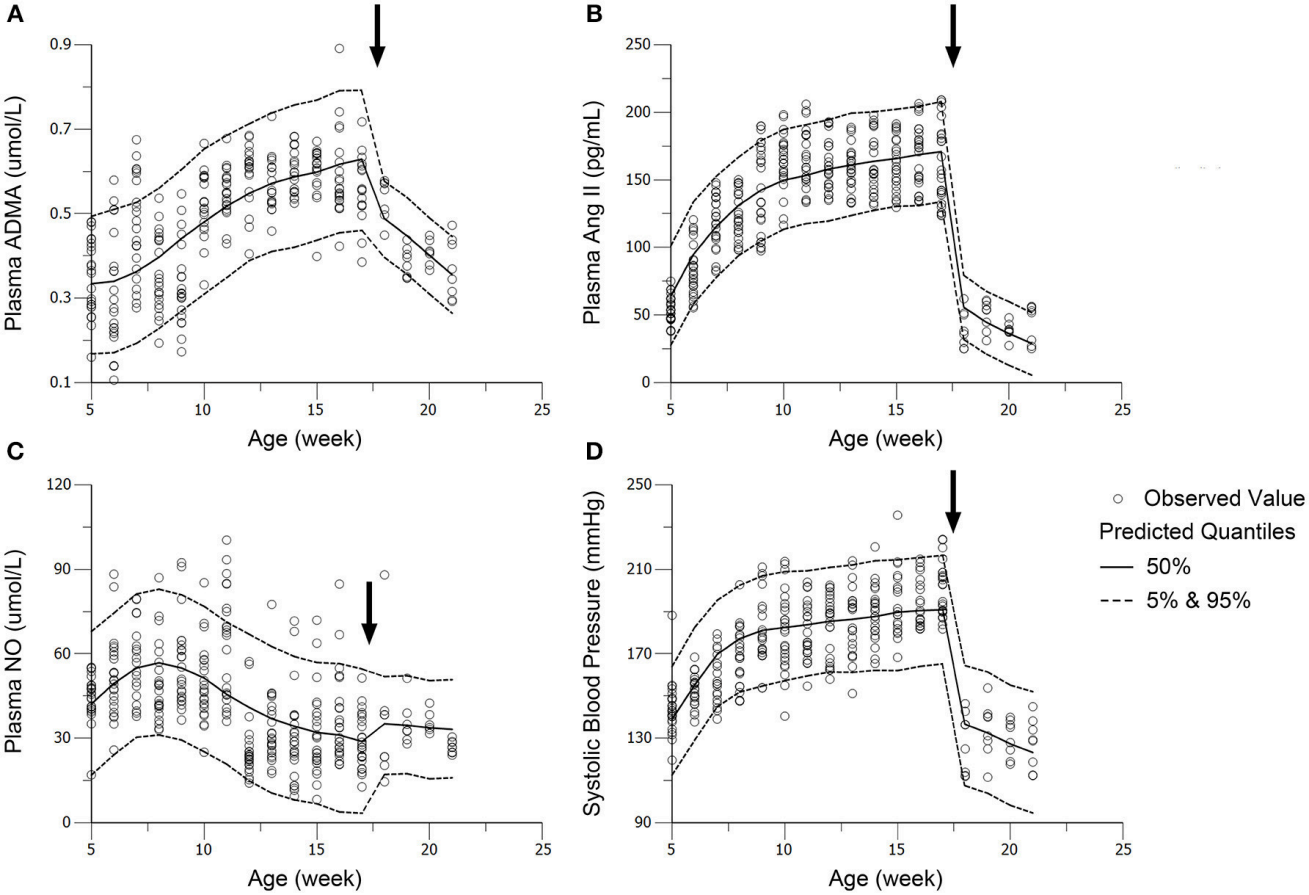
**Visual predictive check (VPC) of models for ADMA (A)**, Ang II **(B)**, NO **(C)**, and SBP **(D)** in the intensive treatment group (receiving ramipril 10 mg/kg^*^day). Parameters (except *DOSE*) in standard treatment groups were fixed in the performance of VPC. The solid circles represent the observed data from intensive treatment group (10 mg/kg). The solid lines represent the 50th percentiles of the 1000 simulations. The dashed lines are the upper (95%) and lower (5%) quantiles of the 1000 simulations. The arrows point to the onset time of treatment.

**Figure 7:**
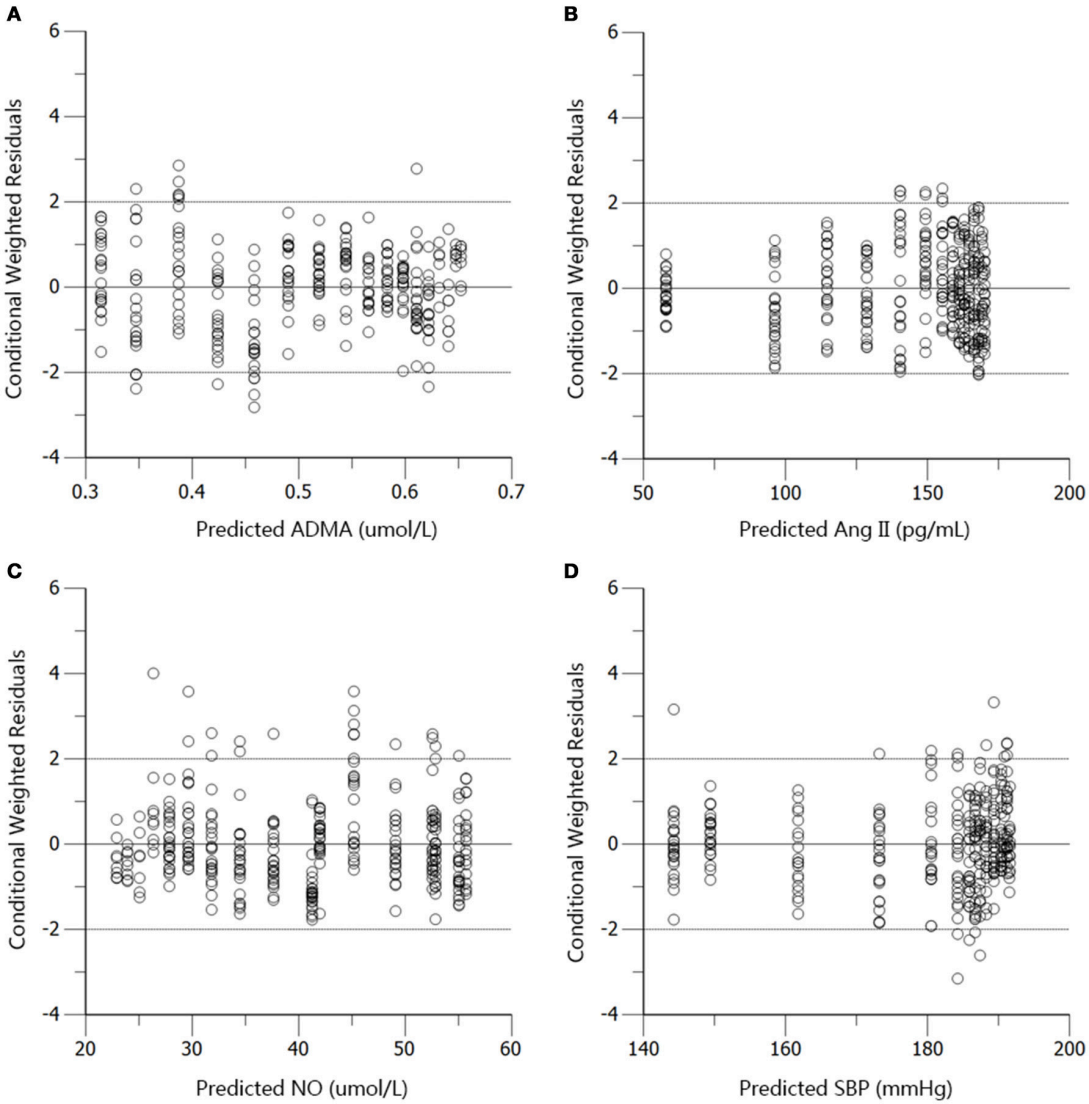
**Conditional weighted residuals (CWRES) vs. predictions (PRED) for ADMA (A)**, Ang II **(B)**, NO **(C)**, and SBP **(D)** in the disease group.

**Figure 8:**
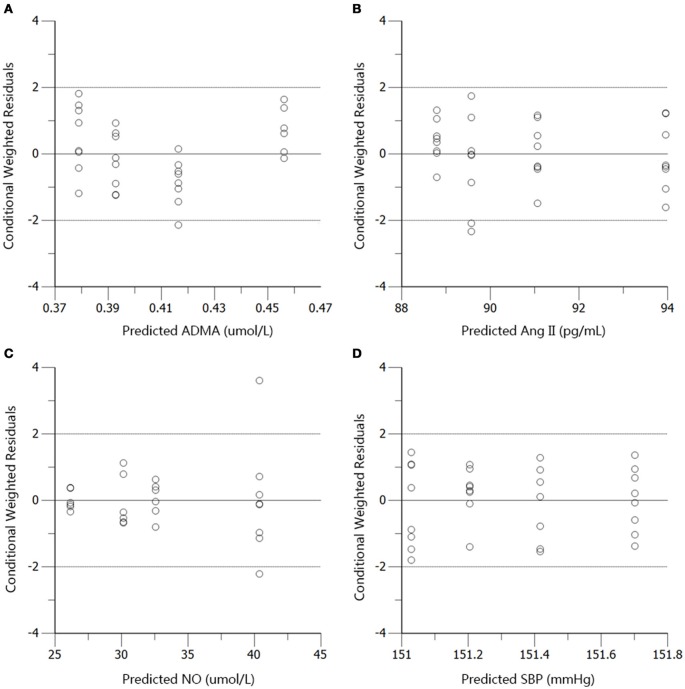
**Conditional weighted residuals (CWRES) vs. predictions (PRED) for ADMA (A)**, Ang II **(B)**, NO **(C)**, and SBP **(D)** in the standard treatment group.

The profiles of SBP, plasma Ang II, ADMA, and NO throughout disease progression were reasonably fitted with our proposed model. The predicted values of SBP, plasma Ang II, ADMA, and NO in disease group (Figure 4) and standard treatment group (Figure 5) were fitted with the mean values from the original data set. Parameter estimates and the optimized transit compartment numbers are listed in Table 1. Parameter estimates obtained from the fit of original mean data were within the mean ± *SD* estimates of the bootstrap replicates. The result of VPC for intensive treatment group has been demonstrated in Figure 6. The observed values are well within the range between 5 and 95 percentiles of 1000 simulated values. The conditional weighted residuals (CWRES) were randomly and homogenously distributed around 0 (Figures 7, 8). The results suggested a reasonable precision in the parameter estimates for the final model.

**Table 1:** **Estimates and definition of parameters of the progression model in three groups**.

**Parameter (Unit)**	**Definition**	**Original dataset**	**Bootstrap dataset**	
			**Mean ±*SD***	**%CV**
*K_*in*_*ANG*_* (pg/mL/week)	Ang II production rate	122.0	115.4±9.3	8.09
*K_*out*_*ANG*_* (1/week)	Ang II output rate	2.111	1.98±0.16	8.369
*K_*in*_*ADMA*_* (umol/L/week)	ADMA production rate	0.1841	0.1855±0.012	6.26
*K_*out*_*ADMA*_* (1/week)	ADMA output rate	0.5854	0.5881±0.043	7.34
*K_*in*_*NO*_* (umol/L/week)	NO production rate	24.02	25.71±2.1	8.22
*K_*out*_*NO*_* (1/week)	NO output rate	0.5721	0.6033±0.046	7.64
*K_*in*_*SBP*_* (1/week)	SBP production rate	0.5075	0.5237±0.045	8.64
*K_*out*_*SBP*_* (1/week)	SBP output rate	0.003520	0.003638±0.00032	8.80
*OX_DDAH* (mL/week/pg)	Disease factor: inhibition of DDAH activity through oxidative effects	0.01668	0.01671±0.0014	8.58
*IN_NOS* (L/week/umolmol)	Disease factor: inhibition of NOS activity by ADMA	0.7085	0.7331±0.053	7.30
*OX_NOS* (mL/week/pg)	Disease factor: inhibition of NOS activity through oxidative effects	0.001971	0.001982±2.1*E*−05	1.08
*ES_*SBP*_* (1/mmHg)	Stimulation of NO production by SBP	0.005028	0.005007±3.8*E*−04	7.63
*ES_*ANG*_* (1/mmHg)	Negative feedback effect of Ang II	0.003453	0.003443±7.8*E*−05	2.26
*VCON* (mL/week/pg)	Vasoconstriction effect of Ang II	0.8718	0.8975±0.048	5.30
*VDIL* (L/week/umolmol)	Vasodilation effect of NO	0.8802	0.8691±0.065	7.52
*EI_*RAMI*_*	Inhibitory effect from ramipril	1.114	1.128±0.088	7.84
*kt*_1_(1/week)	Transduction rate constant	5.348	5.386±0.46	8.49
*kt*_2_(1/week)	Transduction rate constant	0.3388	0.3493±0.018	5.27
*kt*_3_(1/week)	Transduction rate constant	0.4441	0.471±0.047	9.94
*kt*_4_(1/week)	Transduction rate constant	0.03067	0.02937±0.0069	23.5
*kt*_5_(1/week)	Transduction rate constant	3.825	3.7957±0.081	2.14
m	Number of transit compartments		1	
n	Number of transit compartments		1	
o	Number of transit compartments		2	
p	Number of transit compartments		1	
q	Number of transit compartments		1	

### Model simulation

**Figure 9:**
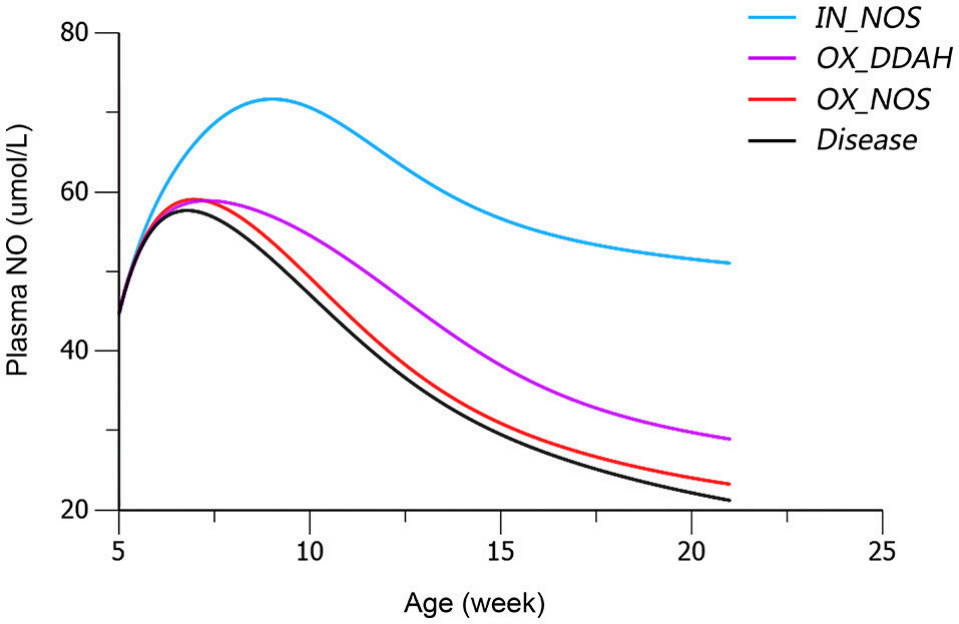
**Simulated plasma nitric oxide (NO) concentrations with the blockade of affection from each of the three disease factors, respectively**. The predicted values of plasma NO in disease group are shown in black line. The predicted values of plasma NO with blockade of disease factor *IN_NOS* [inhibition of NO synthase (NOS) activity by asymmetric dimethylarginine (ADMA)] are shown in blue line. The predicted values of plasma NO with blockade of disease factor *OX_DDAH* (Ang II-mediated inhibition on dimethylarginine dimethylaminohydrolase (DDAH) activity) are shown in purple line. The predicted values of plasma NO with blockade of disease factor *OX_NOS* (inhibition of NOS activity through oxidative effects) are shown in red line. The definitions of disease factors are shown in Table 1 and method.

Based on mean parameter estimates of the model, simulations were performed to predict the fraction of three disease factors contributed to the turnover of plasma NO (Figure 9). Each of the three disease factors (*OX_DDAH, OX_NOS, IN_NOS*) was fixed at 0 for each simulation, assuming the blockade of such disease procedure initiated by the corresponding disease factor. The locations of simulated blockades are shown in Figure 1. NO production was affected by three disease factors with different extensions (Figure 9). The blockade of *IN_NOS* showed the most significant improvement in NO production (Figure 9, blue line). While blocking Ang II-mediated inhibition of dimethylarginine dimethylaminohydrolase (DDAH) activity (*OX_DDAH*) showed much milder effects on NO production (Figure 9, purple line). Compared with them, blocking the direct effect of Ang II-mediated oxidative stress on NOS (*OX_NOS*) has the minimum improvement on NO production (Figure 9, red line). Simulated data indicate that Ang II inhibited NO production mainly through affecting ADMA hydrolysis rather than suppressing NOS activity directly.

## Discussion

Nitric oxide (NO) is regarded as the controller of vascular tone together with vasoconstriction factors, controlling blood pressure. Besides, the abnormality of NO production will result in endothelial dysfunction, leading to various cardiovascular pathologies, like hypertension and atherosclerosis (Bryan, 2006; Rochette et al., 2013). Therefore, modeling and simulating the progression of NO production impairment could provide better understanding of the disease progression in hypertension. On the other hand, oxidative stress has been considered to cause endothelial dysfunction in hypertensive subjects (Schulz et al., 2011). Over produced ROS induced by angiotensin II (Ang II) would suppress the activity of NO synthase (NOS) through oxidizing tetrahydrobiopterin (BH_4_). Besides, dimethylarginine dimethylaminohydrolase (DDAH), the metabolic enzyme of asymmetric dimethylarginine (ADMA), is also sensitive to free radicals (Palm et al., 2007). An enhancement of oxidative stress in SHR has been demonstrated in a variety of researches. In the work of Simao et al, renal H_2_O_2_, NADPH oxidase expression as well as urinary thiobarbituric acid reactive substances (TBARS) was found to increase in SHR (Simao et al., 2011). Increased kidney TBARS was also found in SHR, indicating enhanced oxidative stress (Chandran et al., 2014). Therefore, we incorporated two series of transit compartments in our model, simulating Ang II-mediated oxidative effects on DDAH and NOS activities, triggered by two disease factors (*OX_DDAH* and *OX_NOS*), respectively.

In the work of ND Vaziri et al. (1998), elevated plasma and urinary NO_x_ was observed in 12-week-age SHR, as well as aorta NOS activity. However, in pre-hypertensive SHR (3-week-old), plasma NO_x_ was not significantly elevated compared with Wistar Kyoto (WKY) rats. This might indicate that the elevation of NO production in young SHR is progressive. It is also mentioned that increased NO production during the early stage of hypertension in SHR could not be maintained till the advanced phase of the disease. With progressive endothelial dysfunction, NO production may fall, leading to true NO deficiency in animals with advanced hypertension. In other researches, impaired NO production was also reported preceding the onset of hypertension in SHR (Mokuno et al., 2001). Our results supported the finding of ND Vaziri. In our work, NO production remained at a higher level before the age of 12 weeks in SHR (Figure 3C), indicating elevated NOS activity. Besides, the drop of NO production initiated at the 12th week (Figure 3C) might indicate the start of NOS impairment. Our model captured this procedure (Figure 4C). Model simulations also revealed that three disease factors contribute differently in NO production during disease progression (Figure 9). Firstly, blocking *IN_NOS* showed the most significant improvement in NO production, indicating disease factor *IN_NOS* (inhibition of NOS activity by ADMA) was the major contributor to the inhibition of NO production in disease progression (Figure 9, blue line). Secondly, blocking Ang II-mediated inhibition of DDAH activity (*OX_DDAH*) showed much milder effects on NO production (Figure 9, purple line). According to the mechanism (Figure 1), blockade of *OX_DDAH* could reduce the accumulation of ADMA, decreasing ADMA level. While blocking *IN_NOS* could directly intercept ADMA's inhibition on NO production. Since direct interception of ADMA can obviously provide better improvement on NO production than reducing ADMA accumulation, the results of simulation is reasonable. Finally, blocking the direct effect of Ang II-mediated oxidative stress on NOS (*OX_NOS*) has the minimum improvement on NO production (Figure 9, red line). Compared with the simulated values of blocking *OX_DDAH*, Ang II-mediated oxidative stress might affect NO production mainly through decreasing ADMA elimination instead of inhibiting NOS activity directly. Blocking RAS was proved to protect renal and vascular NOS, increasing NO production (Vaziri et al., 2002; Zhou et al., 2008). In our work, ramipril increased the level of plasma NO in rats from both treatment groups. However, this effect was not enhanced with the increase of dose, which was also reported by Pechánová (2007) and Christian Delles (Delles et al., 2002). This might be explained that blocking RAS could only suppress Ang II-mediated oxidative stress, but other sources of ROS (e.g., xanthine oxidase, mitochondria and cyclooxygenase) might not be attenuated by this action. As a result, the activities of DDAH and NOS might not be better protected from ROS despite a stronger antihypertensive action by a higher dose of ramipril.

## Conclusion

Our work revealed that intensive blood pressure control with ramipril did not bring more benefits to attenuating plasma ADMA and NO in SHR. The proposed model assessed Ang II and ADMA's contribution to NO production impairment in SHR's disease progression. The simulations suggested that Ang II inhibited NO production mainly through affecting ADMA elimination rather than directly affecting NOS activity in hypertension progression of SHR.

## Author contributions

Research design: HW, HH, and XL. Experiment conduction: HW, HJ, XZ, and GR. Modeling and data analysis: HW, HL, and XL. Wrote or contributed to the writing of the manuscript: HW, HH, and XL.

### Conflict of interest statement

The authors declare that the research was conducted in the absence of any commercial or financial relationships that could be construed as a potential conflict of interest.

## Abbreviations

ADMA: asymmetric dimethylarginine
Ang II: angiotensin II
DDAH: dimethylarginine dimethylaminohydrolase
GK rat: Goto-Kakizaki rat
NO: nitric oxide
NOS: nitric oxide synthase
RAS: renin-angiotensin system
ROS: reactive oxygen species
SBP: systolic blood pressure
SHR: spontaneously hypertensive rat.
